# Supplementary material for: Content-rich biological network constructed by mining PubMed abstracts
Source: BMC Bioinformatics. 2004 Oct 8;5:147. doi: 10.1186/1471-2105-5-147 (PMC528731; doi:10.1186/1471-2105-5-147)
Supplement: Additional File 2 — The original results of the above study (non-essential files are deleted to keep the file size under the limit set by BMC bioinformatics). [file 1471-2105-5-147-S2.bz2 › chilibotAdditionalFile2/dip10/733ID11983079E18091/html/FDX_ISCS.html]

 


 **FDX** and **ISCS** 
  
Found 11 abstracts in PubMed, retrieved 10.  
 

 What does Google say? 
 PDF only 
| .edu only 

---

**Interactive relationship** (e.g. stimulation, inhibition, etc)

**Neutral relationship**- In addition, we have identified previously unreported interactions between  **IscS**  and  **Fdx** ,  **IscS**  and ORF3, IscA and HscA, and HscA and  **Fdx** .  Ref: 11983079 J Biochem Tokyo , 2002
- Hyperproduction of recombinant ferredoxins in escherichia coli by coexpression of the ORF1 ORF2  **iscS**  iscU iscA hscB hs cA  **fdx**  ORF3 gene cluster.  Ref: 10393315 J Biochem Tokyo , 1999
- Here we show that cysteine desulfurase  [ **ISCS** ]  encoded by the gene  **iscS**  of E. coli can directly convert the ferredoxin  [ **FDX** ]  dinitrosyl iron complex to the ferredoxin 2Fe 2S  [ **FDX** ]  cluster in the presence of L cysteine in vitro.  Ref: 11825893 J Biol Chem, 2002
- The NifS like protein  **IscS**  was recently reported to mediate the incorporation of iron sulfur clusters into ferredoxin  [ **FDX** ]  in vitro.  Ref: 11355631 Indian J Biochem Biophys, 2000
- The results suggest that cysteine desulfurase  [ **ISCS** ]   **IscS**  together with L cysteine can efficiently repair the nitric oxide modified ferredoxin 2Fe 2S  [ **FDX** ]  cluster.  Ref: 11825893 J Biol Chem, 2002
- Our findings support the conclusion that  **IscS** , via cysteine desulfurase  [ **ISCS** ]  activity, provides the sulfur that is subsequently incorporated into Fe S clusters by assembler machinery comprising of the iscUA hscBA  **fdx**  gene products.  Ref: 11432781 J Biochem Tokyo , 2001
- This apparatus consists of some ten proteins including a cysteine desulfurase  [ **ISCS** ]  producing elemental sulfur for biogenesis, a ferredoxin  [ **FDX** ]  involved in reduction, and two chaperones.  Ref: 10595578 Biol Chem, 1999

**Non-interactive relationship** (e.g. studied together, co-existance, homology, etc.)

- Functional assignment of the ORF2  **iscS**  iscU iscA hscB hscA  **fdx**  ORF3 gene cluster involved in the assembly of Fe S clusters in Escherichia coli.  Ref: 10544286 J Biochem Tokyo , 1999
- We examined the coexpression of the E. coli ORF1 ORF2  **iscS**  iscU iscA hscB hsc A  **fdx**  ORF3 gene cluster, which has recently been suggested to be involved in the formation or repair of Fe S protein Zheng, L.  Ref: 10393315 J Biochem Tokyo , 1999
- In Escherichia coli, genes in the ORF1 ORF2  **iscS**  iscU iscA hscB hsc A  **fdx**  ORF3 cluster the isc gene cluster should be involved in the assembly of the Fe S cluster since its coexpression with the reporter ferredoxin  [ **FDX** ]  Fd dramatically increases the production of holoFd Nakamura, M.  Ref: 10544286 J Biochem Tokyo , 1999
- Thus, the sequential association and dissociation among the  **IscS** , IscU, IscA, HscB, HscA,  **Fdx** , and ORF3 proteins may be a critical process in the assembly of Fe S clusters.  Ref: 11983079 J Biochem Tokyo , 2002
- We also examined coexpression using the  **fdx**  gene in the isc gene cluster as a reporter Fd and identified  **iscS** , hscB, hscA, and ORF3 as being involved in the assembly of the 2Fe 2S cluster in this protein.  Ref: 10544286 J Biochem Tokyo , 1999
- Repair of nitric oxide modified ferredoxin 2Fe 2S  [ **FDX** ]  cluster by cysteine desulfurase  [ **ISCS** ]   **IscS** .  Ref: 11825893 J Biol Chem, 2002
- By coexpressing these plasmids with reporter Fds, we show the  **iscS** , iscA, hscA, and  **fdx**  genes to be required for the assembly of the Fe S clusters.  Ref: 10544286 J Biochem Tokyo , 1999
